# Case report: When Behçet’s disease meets multizonal outer retinopathy and retinal pigment epitheliopathy: longitudinal multimodal imaging of an overlap phenotype

**DOI:** 10.3389/fimmu.2026.1867734

**Published:** 2026-06-12

**Authors:** Jiang Jikuan, Cheng Yizhe, Chen Chunli

**Affiliations:** 1 Beijing Tongren Hospital, Capital Medical University, Beijing Tongren Eye Center, Beijing Ophthalmology and Visual Science Key Laboratory, Beijing, China; 2 State Key Laboratory of Ophthalmology, Zhongshan Ophthalmic Center, Sun Yat-sen University, Guangdong Provincial Key Laboratory of Ophthalmology and Visual Science, Guangdong Provincial Clinical Research Center for Ocular Diseases, Guangzhou, China

**Keywords:** Behçet’s disease, case report, multimodal imaging, multizonal outer retinopathy and retinal pigment epitheliopathy, retinal vasculitis

## Abstract

**Purpose:**

To report a rare case of overlapping features of Behçet’s uveitis and multizonal outer retinopathy and retinal pigment epitheliopathy (MORR), documented by longitudinal multimodal imaging.

**Methods:**

A single case report.

**Results:**

A 37-year-old male presented with unilateral, painless, progressive vision loss and a history of recurrent oral ulcers. Multimodal imaging with longitudinal follow-up documented bilateral, progressive outer retinopathy concurrent with retinal vasculitis. These features characterize an acute progressive episode not previously described.

**Conclusion:**

This case highlights the importance of multimodal imaging and reminds clinicians to remain vigilant to possible concurrent outer retinopathy in Behçet’s uveitis. Furthermore, given the favorable treatment response observed here, this case also offers a reference for the therapeutic strategy of MORR.

## 1 Introduction

Behçet’s disease is a chronic, relapsing systemic vasculitis characterized by recurrent oral and genital ulcers, cutaneous lesions, and ocular inflammation (1). Ocular involvement can manifest as posterior uveitis with retinal vasculitis, which is a major cause of vision loss (2). Beyond vasculitic uveitis, immune-mediated retinal inflammatory disorders can also predominantly involve the outer retina and retinal pigment epithelium (RPE), leading to progressive photoreceptor dysfunction. Within this spectrum, acute zonal occult outer retinopathy (AZOOR) and its variant, multizonal outer retinopathy and retinal pigment epitheliopathy (MORR), are characterized by dysfunction of the photoreceptor and retinal pigment epithelium layers, often classified as autoimmune or inflammatory retinopathies (3).

While the ocular manifestations of Behçet’s disease are well-established, its co-occurrence with outer retinopathies such as AZOOR or MORR (a variant of AZOOR) is rarely documented. Whether these diseases share clinical or pathophysiological overlap remains unknown.

We report a young man who presented with unilateral, painless, progressive vision loss. Multimodal imaging revealed features consistent with both Behçet-associated retinal vasculitis and MORR. This case highlights the potential association between these two distinct inflammatory entities and provides insights into the therapeutic response and clinical course of this complex presentation.

## 2 Results

A 37-year-old male presented with painless, progressive visual loss in his right eye, which was frequently exacerbated by mental fatigue. He has bilateral high myopia. Notably, he also reported recurrent oral aphthous ulcers, occurring approximately 6 to 7 times per year. History of genital ulcers was denied.

Best-corrected visual acuity (BCVA) was 20/66 in the right eye and 20/25 in the left eye. Slit-lamp examination showed a quiet anterior chamber, with a few inflammatory cells detected in the vitreous cavity of both eyes alongside vitreous opacities. Fundus examination revealed a bilateral tessellated fundus with subtle peripapillary pigmentary changes. No abnormalities were noted at the macula. The optic disc had sharp margins with a normal cup-to-disc ratio. The peripheral retina was flat and intact without holes, tears, or degenerative changes.

**Figure 1:**
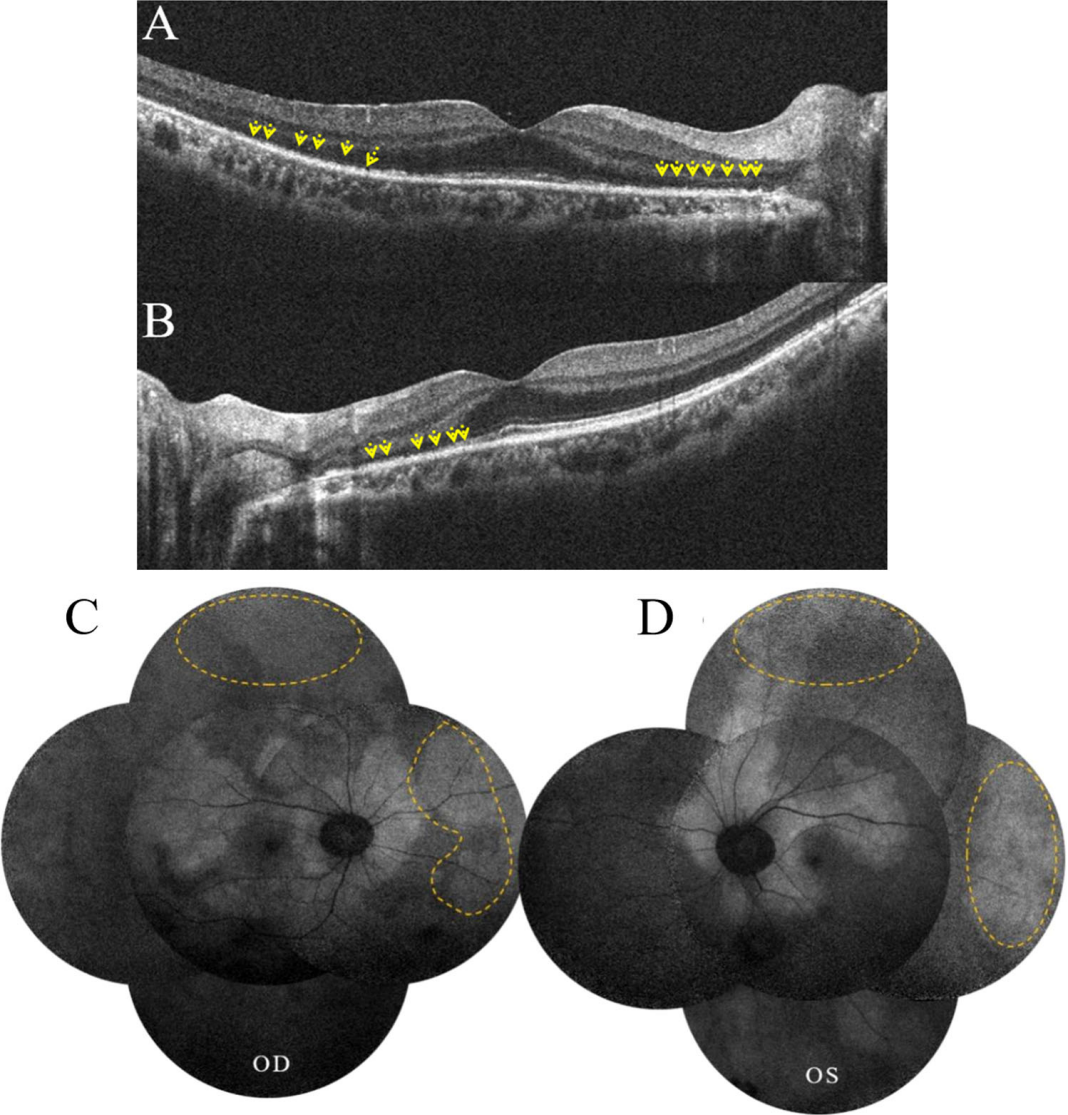
**(A, B)** SD-OCT images. Yellow arrows indicate the disruption of the outer retinal bands (ellipsoid zone and interdigitation zone). **(C, D)** FAF image demonstrates bilateral, multifocal retinal involvement. Areas of hyperfluorescence in the peripheral retina are outlined with yellow dashed circles.

Spectral-domain optical coherence tomography (SD-OCT) revealed focal loss of the outer retinal layers, primarily around the optic disc, extending from the external limiting membrane to the interdigitation zone. Additional involvement was the disruption of the photoreceptors in the macula (**Figures 1A, B**).

Fundus autofluorescence (FAF) imaging revealed the patchy hyperautofluorescence around the optic disc, with a map-like distribution extending widely across the posterior pole. Multifocal areas of hyperautofluorescence in the peripheral retina were observed, which were not connected to the peripapillary hyperautofluorescent lesions. (**Figures 1C, D**).

**Figure 2:**
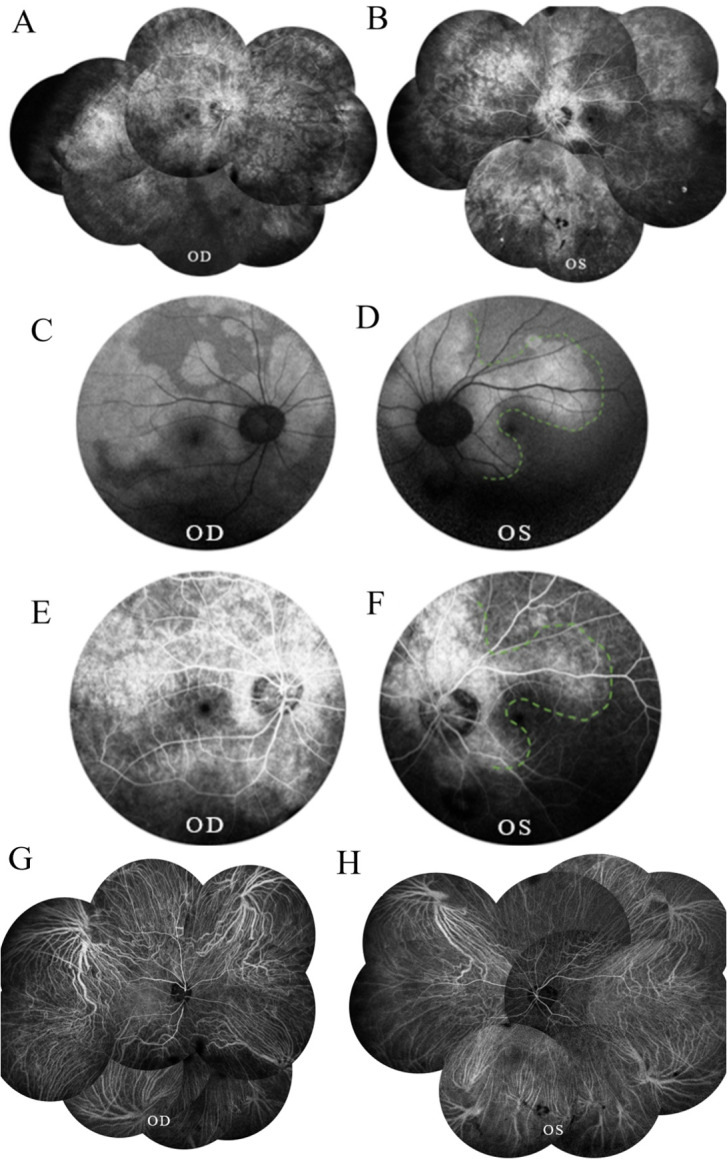
**(A, B)** FFA demonstrates extensive involvement of bilateral retinal vasculature. **(C–F)** FAF and FFA images of the posterior pole. Green dashed circles highlight the overlap areas of vascular leakage on FFA and corresponding hyperfluorescent regions on FAF. **(G, H)** ICGA image shows no abnormalities in the choroidal circulation.

On fluorescein angiography (FFA), bilateral, diffuse perivascular leakage with a fern-like pattern was observed across the entire retina (**Figures 2A–F**). The area of most prominent vascular leakage on FFA partially corresponded to the hyperautofluorescent areas observed on FAF. In contrast, ICGA revealed no abnormal findings (**Figures 2G, H**).

Serological investigations were negative for syphilis, tuberculosis, toxoplasmosis, and HIV. Vasculitis-associated antibodies were within the normal reference range. Biochemistry was unremarkable, including electrolytes, liver and renal function and fasting glucose. Inflammatory markers including ESR, CRP were normal. HLA-B51 tested negative.

**Figure 3:**
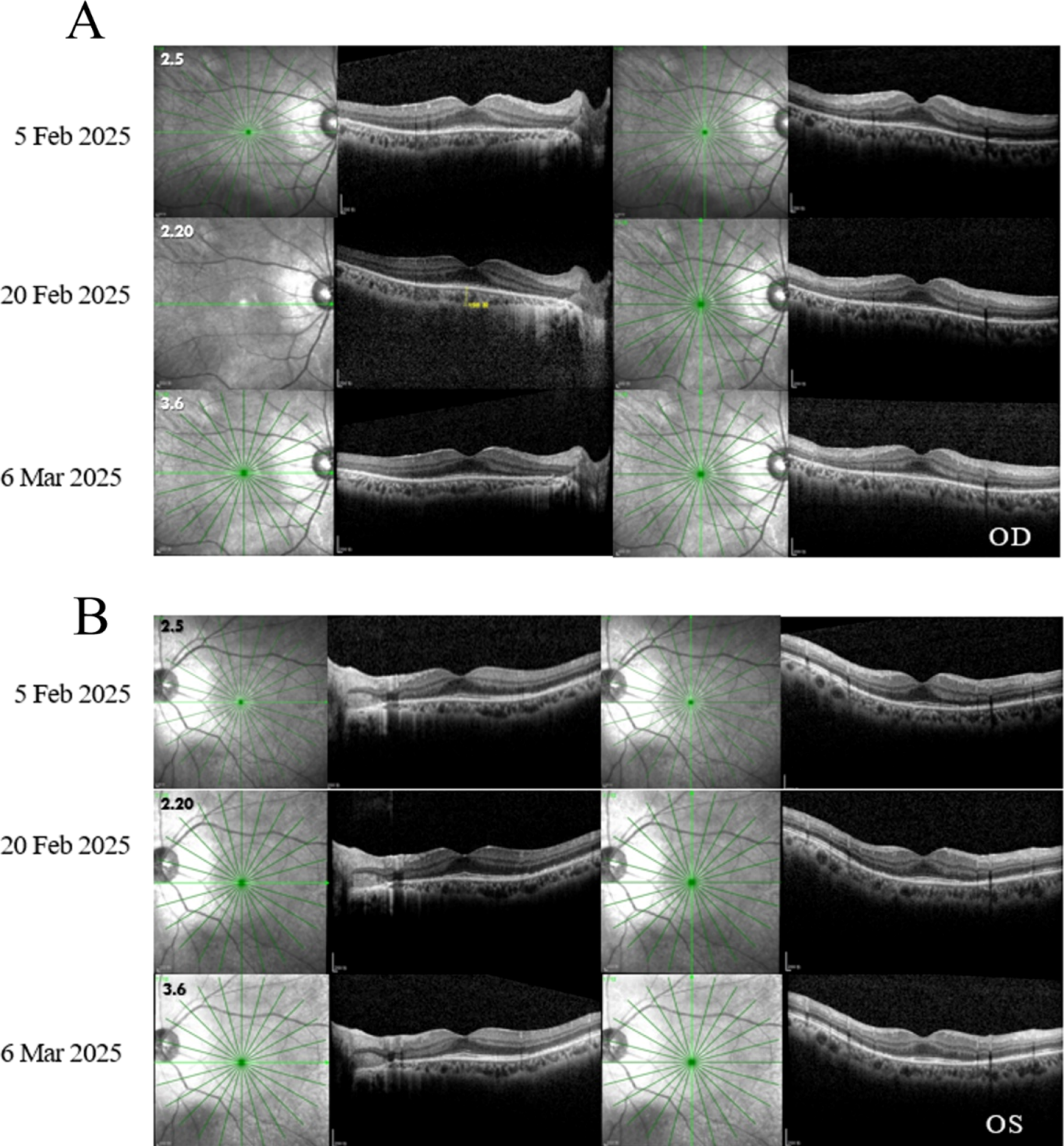
**(A, B)** SD-OCT scans of the macula in horizontal and vertical orientations showed no significant progression in one-month follow-up.

**Figure 4:**
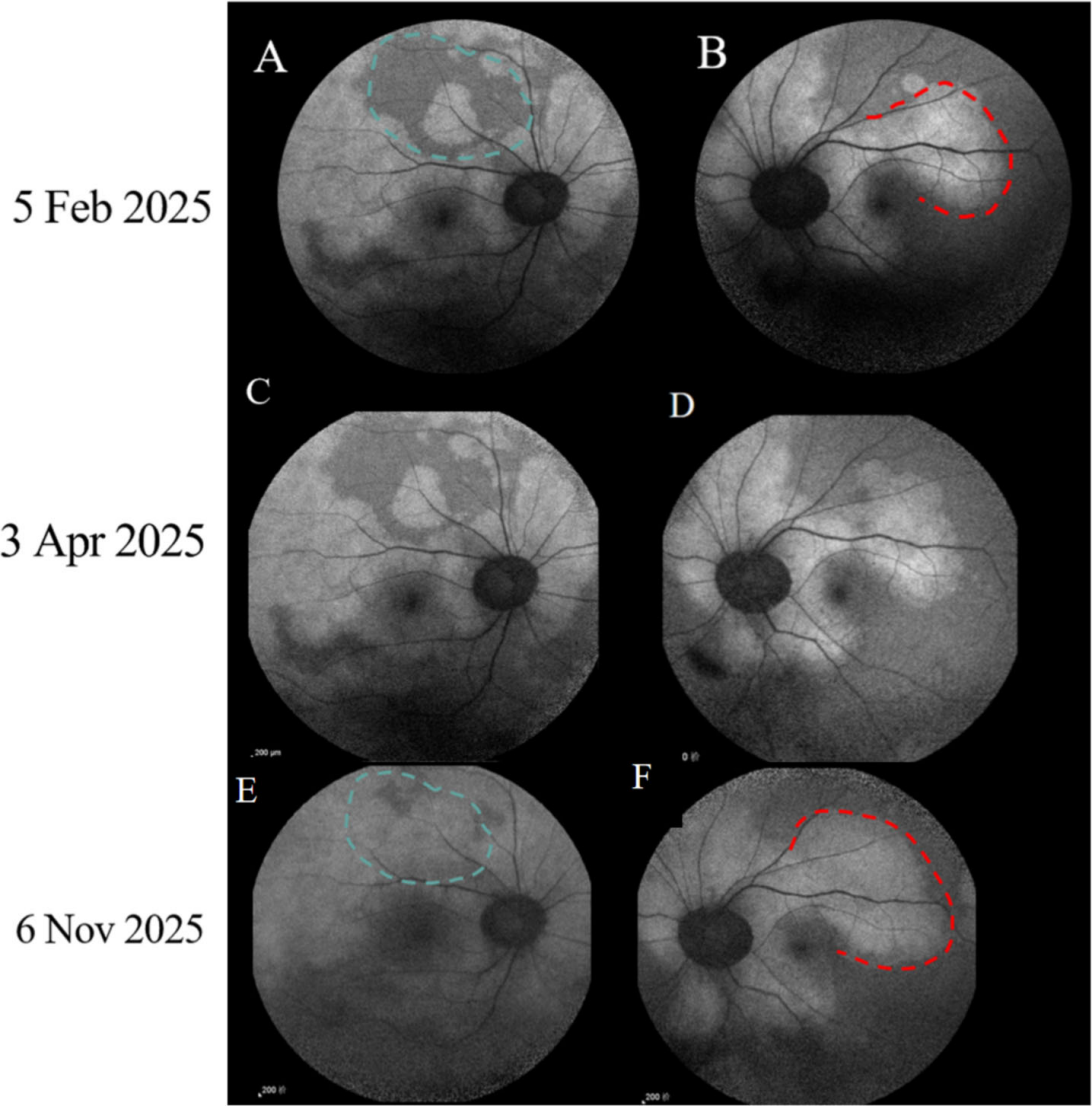
**(A–D)** FAF imaging showed no significant expansion in the hyperautofluorescent lesions in two-months follow-up. **(E, F)** FAF images at 9-month follow-up showing disease progression: a tendency of lesions to merge in the right eye (blue circles) and peripheral expansion with a ‘grass-fire’ pattern of spread in the left eye (red circles).

Based on the findings, a diagnosis of MORR (a variant of AZOOR) complicated by Behcet’s uveitis was established. Considering the acute progression and widespread retinal involvement, systemic treatment was commenced with oral corticosteroids (1 mg/kg) combined with the immunosuppressant cyclosporine. At the three-month follow-up after treatment initiation, the lesions remained largely stable on OCT (**Figures 3A, B**), and FAF (**Figures 4A–D**), indicating no evident progression. At the 9-month follow-up, when the oral corticosteroid was tapered to a maintenance dose, subtle progression was observed (**Figures 4E, F**). Best-corrected visual acuity remained at 20/66 in the right eye and 20/25 in the left eye.

## 3 Discussion

We present a case of a 37-year-old male with painless visual loss and longitudinal multimodal imaging findings. The retinal angiographic features and oral aphthosis were consistent with Behçet disease. In parallel, the pattern of outer retinal and RPE involvement followed an AZOOR-spectrum phenotype. Based on the work by Ramtohul et al. (4), we contend that the outer retinal changes in this patient are more consistent with MORR than AZOOR. Several outer retinopathies were considered in the differential diagnosis. Acute annular outer retinopathy was considered less likely because the lesions in our patient were bilateral and multizonal, involving both the peripapillary/posterior pole region and noncontiguous peripheral retina, rather than a predominantly unilateral annular peripapillary lesion (5). Autoimmune retinopathy and paraneoplastic retinopathy were also considered; however, these entities usually present with otherwise unexplained photoreceptor dysfunction, often with electroretinographic abnormalities and without prominent intraocular inflammation or retinal vasculitis (6, 7). In contrast, our patient showed bilateral diffuse retinal vascular leakage, mild vitreous inflammation, and recurrent oral aphthous ulcers fulfilling the clinical criteria for Behçet’s disease.

Behcet disease is a rare, chronic systemic vasculitis that can affect multiple organ systems, classically characterized by recurrent oral and genital aphthous ulcers, ocular inflammation most notably in the form of posterior uveitis or retinal vasculitis, and polymorphic skin lesions (8). Despite its systemic nature, ocular involvement occurs in approximately 50% to 70% of patients (9). The predominant ocular manifestation of Behcet disease is uveitis. Posterior segment involvement commonly presents with vitreous cells and haze, retinal vasculitis, retinitis, and optic neuritis. Retinal vasculitis in Behcet’s disease is considered the core phenotype (10). It typically involves small and medium-sized arteries and veins, leading to vascular occlusion, exudation, and vitreous hemorrhage (8). Additionally, recurrent or persistent vasculitis may lead to retinal ischemia and secondary complications (11–13). According to the International Criteria for Behçet’s Disease (ICBD), our patient had typical ocular involvement (2 points) and recurrent, painless oral ulcers occurring more than three times per year (2 points), yielding a total score of ≥4 points and meeting the diagnostic threshold for Behçet’s disease (14).

Nevertheless, cases of concurrent outer retinal changes in Behcet’s uveitis have been rarely reported. The working diagnosis for this case was AZOOR, a rare inflammatory disorder characterized by dysfunction and loss of the outer retinal layers (15), first described by Gass in 1993. Ramtohul et al. described multizonal outer retinopathy and retinal pigment epitheliopathy (MORR), recognized as a variant of AZOOR, which is peripapillary and far-peripheral annular involvement with a stereotyped longitudinal evolution on fundus autofluorescence, including an expanding hypoautofluorescent core bordered by a demarcation line with fringe-like hyperautofluorescent features (4).

Several features help distinguish MORR from classic AZOOR. Classic AZOOR typically presents with a single or limited zone of outer retinal dysfunction and is often unilateral at onset, with lesions that may be occult or show minimal early funduscopic changes. In contrast, MORR tends to demonstrate multizonal and bilateral involvement from the early stages, preferentially affecting the peripapillary region and far peripheral retina. Funduscopic abnormalities, such as retinal pigment epithelium mottling, atrophy, or pigment clumping, are also more commonly visible in MORR (3). On fundus autofluorescence, MORR follows a more stereotyped pattern of progression, with an expanding hypoautofluorescent core bordered by a fringe-like hyperautofluorescent line, a finding less consistently described in classic AZOOR (4). In our patient, the presence of additional lesions not contiguous with the peripapillary changes further supported a MORR-like distribution.

An important feature of this case is the discrepancy between bilateral imaging abnormalities and asymmetric visual function. Although the patient presented with clinically apparent visual loss in the right eye, the left eye already showed multimodal imaging evidence of outer retinal/RPE involvement and retinal vasculitis at baseline, despite relatively preserved BCVA. Because no multimodal imaging was available before presentation, the exact inter-eye chronology cannot be reconstructed. Thus, the bilateral findings may represent either phase-shifted involvement between the two eyes or synchronous bilateral disease with asymmetric structure-function impact. A recently published case report of MORR with chronologically divergent bilateral involvement further supports the concept that MORR may show asymmetric inter-eye evolution over time (16). The observation also emphasizes that, in MORR overlapping with Behçet’s disease, fellow-eye involvement may be clinically silent or functionally mild at an early stage, supporting the need for bilateral multimodal imaging even when visual symptoms are unilateral.

The pathogenesis of Behcet syndrome is not fully elucidated, it is sustained by the relationship between infectious agents, genetic, immune system dysfunction and environmental factors. Neutrophil hyperreactivity (17), heightened production of pro-inflammatory cytokines (18, 19), together with a Th1/Th17-skewed milieu (20, 21), promote endothelial activation and vascular inflammation, predisposing to breakdown of the blood–retina barrier during ocular inflammation. In contrast, AZOOR-spectrum disorders are generally considered as an autoimmune/inflammatory disease, targeting the photoreceptor–RPE complex (3). Genetic susceptibility and environmental triggers play a critical role in the pathogenesis of AZOOR/MORR (22). Another hypothesis proposes that AZOOR/MORR may be initiated by virus entering the retina via the peripapillary region or the ora serrata. The virus is thought to spread among photoreceptors, subsequently triggering a damaging immune response against them (22, 23). We hypothesize a potential pathogenic crossover between Behcet’s uveitis and AZOOR/MORR. In our patient, angiographic leakage and mild vitreous inflammation are most parsimoniously attributed to Behçet-related retinal vasculitis, whereas the longitudinal FAF/OCT pattern is more compatible with a MORR-like outer retinopathy based on the imaging framework proposed by Ramtohul et al (4). We speculate a two-step, two-compartment interaction: systemic immune activation and vasculitis-associated barrier disruption may facilitate local exposure of outer-retinal/RPE antigens and amplify bystander inflammatory injury, thereby precipitating or accelerating an AZOOR-spectrum process in predisposed individuals. Notably, we observed a partial topographic overlap between the areas of most prominent vascular leakage and the zones affected by MORR (**Figures 2C–F**). We suppose that the immune dysregulation of active Behcet’s disease confers a heightened vulnerability, and environmental triggers might more readily induce AZOOR/MORR in genetically predisposed individuals.

There is no consensus on the efficacy of anti-inflammatory drugs for AZOOR-spectrum disorders. Early administration of corticosteroids is thought to be beneficial for arresting disease progression in AZOOR/MORR. Studies have reported that early initiation of corticosteroid treatment can lead to recovery of the ellipsoid zone integrity in the macula (24). However, in advanced stages of the disease, the outer retinal damage becomes irreversible. And anti-inflammatory therapy is likely to be of minimal to no efficacy (25). Despite viral infection being proposed as an etiologic hypothesis, antiviral therapy has not demonstrated a clear benefit in AZOOR. Compared to AZOOR, MORR typically progresses more rapidly and involves more extensive areas of the retina. Oral corticosteroid therapy exerts a stabilizing effect in the early stages but does not completely halt further lesion progression (4, 26). During the maintenance treatment following the acute phase, immunosuppressive therapy tends to show a favorable response in managing MORR (26). In our case, combined oral corticosteroids and immunosuppressants in this case seem to halt the acute exacerbation, which supported the initial therapeutic strategy. However, the observation of subtle lesion progression following corticosteroid tapering indicates that the optimal timing and rate of dose reduction in MORR require further optimization. Local corticosteroid therapy, particularly intravitreal dexamethasone implant, has been described as a potential alternative or adjunctive treatment in selected AZOOR cases, with reported improvement or stabilization of disease activity (27, 28). However, in the present case, systemic treatment was favored because the patient had bilateral involvement with diffuse retinal vascular leakage and mild vitreous inflammation, suggesting concomitant Behçet-related posterior segment inflammation requiring systemic immunosuppression.

This report presents a rare co-occurrence of Behcet’s uveitis and MORR. It serves as a critical reminder that outer retinopathies like MORR may not exist in isolation and may be facilitated or unmasked within the dysregulated immune environment of a systemic disorder.

## Data Availability

The original contributions presented in the study are included in the article/supplementary material. Further inquiries can be directed to the corresponding authors.
